# Development and validation of a cognitive dysfunction risk prediction model for the abdominal obesity population

**DOI:** 10.3389/fendo.2024.1290286

**Published:** 2024-02-28

**Authors:** Chun Lei, Gangjie Wu, Yan Cui, Hui Xia, Jianbing Chen, Xiaoyao Zhan, Yanlan Lv, Meng Li, Ronghua Zhang, Xiaofeng Zhu

**Affiliations:** ^1^ General Practice, The First Affiliated Hospital of Jinan University, Guangzhou, Guangdong, China; ^2^ School of Traditional Chinese Medicine, Jinan University, Guangzhou, Guangdong, China; ^3^ College of Pharmacy, Jinan University, Guangzhou, Guangdong, China; ^4^ Cancer Research Institution, Jinan University, Guangzhou, Guangdong, China; ^5^ Guangdong Provincial Key Laboratory of Traditional Chinese Medicine Informatization, Jinan University, Guangzhou, Guangdong, China; ^6^ Traditional Chinese Medicine Department, The First Affiliated Hospital of Jinan University, Guangzhou, Guangdong, China

**Keywords:** abdominal obesity, cognitive dysfunction, NHANES, risk factor, nomogram

## Abstract

**Objectives:**

This study was aimed to develop a nomogram that can accurately predict the likelihood of cognitive dysfunction in individuals with abdominal obesity by utilizing various predictor factors.

**Methods:**

A total of 1490 cases of abdominal obesity were randomly selected from the National Health and Nutrition Examination Survey (NHANES) database for the years 2011–2014. The diagnostic criteria for abdominal obesity were as follows: waist size ≥ 102 cm for men and waist size ≥ 88 cm for women, and cognitive function was assessed by Consortium to Establish a Registry for Alzheimer’s Disease (CERAD), Word Learning subtest, Delayed Word Recall Test, Animal Fluency Test (AFT), and Digit Symbol Substitution Test (DSST). The cases were divided into two sets: a training set consisting of 1043 cases (70%) and a validation set consisting of 447 cases (30%). To create the model nomogram, multifactor logistic regression models were constructed based on the selected predictors identified through LASSO regression analysis. The model’s performance was assessed using several metrics, including the consistency index (C-index), the area under the receiver operating characteristic (ROC) curve (AUC), calibration curves, and decision curve analysis (DCA) to assess the clinical benefit of the model.

**Results:**

The multivariate logistic regression analysis revealed that age, sex, education level, 24-hour total fat intake, red blood cell folate concentration, depression, and moderate work activity were significant predictors of cognitive dysfunction in individuals with abdominal obesity (*p* < 0.05). These predictors were incorporated into the nomogram. The C-indices for the training and validation sets were 0.814 (95% CI: 0.875-0.842) and 0.805 (95% CI: 0.758-0.851), respectively. The corresponding AUC values were 0.814 (95% CI: 0.875-0.842) and 0.795 (95% CI: 0.753-0.847). The calibration curves demonstrated a satisfactory level of agreement between the nomogram model and the observed data. The DCA indicated that early intervention for at-risk populations would provide a net benefit, as indicated by the line graph.

**Conclusion:**

Age, sex, education level, 24-hour total fat intake, red blood cell folate concentration, depression, and moderate work activity were identified as predictive factors for cognitive dysfunction in individuals with abdominal obesity. In conclusion, the nomogram model developed in this study can effectively predict the clinical risk of cognitive dysfunction in individuals with abdominal obesity.

## 1 Introduction

According to the latest World Obesity Map 2023, the whole prevalence of overweight and obesity is projected to reach 51% by 2035, affecting over 4 billion individuals in the world. This upward trend is attributed to modifications in dietary habits and lifestyle choices (1). The consequences of obesity extend beyond personal health, posing significant challenges to public health and economic productivity (2). [Urgent measures are required to address this growing concern and alleviate its burden on individuals, communities, and healthcare systems.

Cognitive functioning encompasses executive functioning, memory, perceptual-motor functioning, verbal functioning, complex attention, and social cognitive functioning. Cognitive dysfunction, which refers to impairment in one or more aspects of cognitive function, has become a social burden and significantly diminishes people’s life experiences (3). In China, there are studies on the risk factors related to cognitive dysfunction. Research by Huilian Duan et al. (4) has shown that unhealthy lifestyles, apolipoprotein E (APOE) ϵ4 genotype, and methyltetrahydrofolate reductase (MTHFR) TT genotype are significantly associated with cognitive dysfunction in Chinese elderly people. Among lifestyle factors, healthy diet and physical exercise play a crucial role in preventing cognitive decline. In a longitudinal study (5) in China, age, BMI, blood pressure, cholesterol, and depression were identified as important predictors of cognitive dysfunction. However, there has been limited research in China investigating the relevant factors influencing cognitive dysfunction in the population affected by abdominal obesity. Research from Europe indicates that obesity affects cognitive abilities such as working memory, language, and executive function (6). A meta-analysis revealed a clear association between abdominal obesity and cognitive dysfunction, with abdominal obesity potentially increasing the likelihood of cognitive impairment (7). Mendelian randomization studies have established a causal link between abdominal obesity and cognitive performance, demonstrating that abdominal obesity negatively impacts cognitive function (8). Understanding these risk factors could enable the identification of high-risk groups at an earlier stage and facilitate the assessment of disease severity and prognosis.

A Nomogram is a tool that calculates a total score by summing up the scores of all Influencing factors. It utilizes these scores to predict the probability of a clinical event occurrence, offering individualized analysis and prediction. Taking into account a number of risk factors, the nomogram can aid in diagnosing or predicting the prognosis of diseases (9). Prior research has not explored risk prediction models specifically associated with cognitive dysfunction and abdominal obesity. Our objective is to identify risk factors for cognitive dysfunction in individuals with abdominal obesity and incorporate them into a nomogram prediction model. This model will help clinicians in early diagnosis, as well as the development of personalized preventive measures and treatment plans.

## 2 Materials and methods

### 2.1 Study design and participants

The data for this study was obtained from the National Health and Nutrition Examination Survey (NHANES) website. NHANES is a comprehensive and nationally representative survey conducted to evaluate and understand the health, well-being, and nutrition of individuals residing in America. It collects data through various methods, including interviews, physical examinations, and laboratory tests, aiming to provide a comprehensive understanding of the health and nutritional characteristics of the population. The survey can be accessed online at https://wwwn.cdc.gov/nchs/nhanes/. For our analysis, we selected the data from 2011 to 2014, which included 19,931 individuals in total.

Sampling method: The NHANES sample design consists of a multiyear, stratified, clustered four-stage sample, with data released on a 2-year cycle, and the four-stage sample is divided primarily into (a) primary sampling units (PSUs) (counties, groups of areas within counties, or combinations of adjacent counties), (b) segments (census tracts or combinations of tracts) within PSUs, (c) dwelling units (DUs) (households) within segments, and (d) individuals within households. The sample represents the noninstitutionalized civilian population residing in the 50 states and the District of Columbia. The specific NHANES sample design, including specifications for clustering, stratification, and oversampling population subgroups, changed over time. Specific methods are available on the NHANES database website. (https://wwwn.cdc.gov/nchs/nhanes/).

Participants who met the diagnostic criteria for abdominal obesity in NHANES from 2011 to 2014 were included in this study. Waist circumference has been identified as a reliable indicator of visceral fat accumulation and adverse metabolic characteristics (10), hence we used waist circumference as a diagnostic measure for abdominal obesity. Inclusion criteria: waist size ≥ 102 cm for men and waist size ≥ 88 cm for women (11). Exclusion criteria: Participants with missing information on cognitive function and covariates.

### 2.2 Ethics statement

Participants who were included in the NHANES database were required to sign an informed consent form. This form has been reviewed and approved by the National Center for Health Statistics Ethics Review Board.

### 2.3 Data selection and measurements

NHANES administered a battery of cognitive performance assessments to participants from 2011 to 2014. This battery of tests included the Consortium to Establish a Registry for Alzheimer’s Disease (CERAD), Word Learning subtest, Delayed Word Recall Test, Animal Fluency Test (AFT), and Digit Symbol Substitution Test (DSST) (12). These tests have been commonly used in large-scale screenings and clinical investigations aimed at assessing the cognitive abilities of older individuals (13–15). The overall score for the CERAD exam was calculated using three Word Learning subtests and a delayed memory test.

The criteria for cognitive dysfunction were based on previous studies (16), which averaged Z-scores for CERAD, AFT, and DSST to generate a composite Z score representing overall cognitive performance. The Z-score formula was as follows: Z = (x-u)/σ, where x represented the test score for each participant, u referred to the mean test score across all participants, and σ referred to the standard deviation of the test scores across participants. Cognitive dysfunction was defined as a Z-score below the 25th percentile, corresponding to a value of -0.56 in our sample.

We selected several potential predictors that may have an impact on cognitive development based on contemporary clinical practice and relevant academic research (17, 18). These predictors included sociodemographic characteristics, lifestyle factors (such as 24-hour nutrient intake, physical activity intensity, sleep time, smoking, etc.), anthropometric variables, laboratory examination variables, and disease status.

Sociodemographic characteristics of the participants included the age, sex, race, education, and marital status. The 24-hour total nutrient intake data were obtained through a 24-hour dietary recall method conducted by two nutritionists. Dietary review interviews took place at a mobile physical examination center, and the calorie and nutrient content of each food and drink were calculated based on the quantity and corresponding nutrients reported by the United States Department of Agriculture. The following nutrient intake variables were included in the research: total energy (kcal), protein (g), carbohydrate (g), dietary fiber (g), fat (g), and alcohol intake (g). Physical activity intensity included vigorous work activity, moderate work activity, vigorous recreational activity, moderate recreational activity, and sedentary minutes. Work and recreational activities were considered if engaged in for more than ten minutes. Sleep time referred to the number of hours slept on weekday nights. A respondent was considered a smoker if they had smoked more than 100 cigarettes in their lifetime. Anthropometric variables included body mass index (BMI) calculated as kg/m². The laboratory tests included red blood cell folate concentration (RBC folate) (nmol/L), glycosylated hemoglobin (%), plasma albumin (g/L), and serum creatinine (mmol/L). Prevalence of hypertension, diabetes, asthma, arthritis, gout, angina, stroke, liver disease, and depression were also considered. Depression was assessed using the Patient Health Questionnaire-9 (PHQ-9), with a score ≥ 5 indicating depression (19). The remaining chronic diseases were based on questionnaire survey data.

### 2.4 Statistical analysis

R software (version 4.2.3) was used for statistical analysis. Shapiro-Wilk tests were performed to check for normal distribution of continuous data. Independent samples t-tests were used to evaluate differences between two normally distributed datasets, and the variables were described with mean ± standard deviation (SD). Mann-Whitney U test was used to analyze differences between the two datasets without normal distribution, and the variables were described with the median and interquartile range (IQR). Meanwhile, chi-square test or Fisher’s exact test was utilized for comparison of categorical variables. Besides, the variables were described with percentages.

The data was randomized into either training set (n = 1043) or validation set (n = 447) using a 7:3 ratio. Lasso regression analysis (20) was applied to the training sample data to select predictors of cognitive dysfunction in abdominal obesity, and the appropriate lambda (λ) value was determined through 10-fold cross-validation. The selected variables were then incorporated into multifactor logistic regression analysis to further filter the variables. The predicted variables with *p* values < 0.05 were incorporated into the nomogram model.

Once the nomogram was constructed, we assessed its performance using various metrics. The consistency index (C-index), the area under the receiver operating characteristic (ROC) curve (AUC), and calibration curves were used to evaluate the predictive ability. Decision curve analysis (DCA) was adopted to assess the clinical applicability of the nomogram.

## 3 Results

### 3.1 Baseline characteristics

Between 2011 and 2014, NHANES included a total of 6,222 individuals with abdominal obesity. After excluding participants with missing information on cognitive dysfunction and covariates, 1,490 participants in total were included in the study. The baseline characteristics of these subjects are presented in **Table 1**. Among them, 882 (59.19%) were female and 608 (40.81%) were male. Approximately 25.23% of the participants reported cognitive dysfunction. Randomly chosen participants with abdominal obesity were split into a training group (n=1,043) and a validation group (n=447).

**Table 1:** Baseline characteristics of the study population.

Variable	Total (n=1490)	Non-cognitive dysfunction (n=1114)	cognitive dysfunction (n=376)	P-value
Age (year)	68.00[63.00, 75.00]	68.00[63.00, 73.00]	71.00[65.75, 78.00]	<0.001
Sex (%)				
Male	608(40.81)	423(37.97)	185(49.20)	
female	882(59.19)	691(62.03)	191(50.80)	0.001
Race (%)				
Mexican American	131(8.79)	85(7.63)	46(12.24)	
Other Hispanic	142(9.53)	80(7.18)	62(16.49)	
Non-Hispanic White	811(54.43)	668(59.96)	143(38.03)	
Non-Hispanic Black	343(23.02)	230(20.65)	113(30.05)	
Other Race	63(4.23)	51(4.58)	12(3.19)	<0.001
Education (%)				
Less than 9th grade	140(9.40)	48(4.31)	92(24.47)	
9-11th grade	208(13.96)	114(10.23)	94(25.00)	
High school graduate	380(25.50)	279(25.04)	101(26.86)	
Some college graduate	459(30.81)	399(35.82)	60(15.96)	
College graduate Or above	303(20.33)	274(24.60)	29(7.71)	<0.001
Marital (%)				
Married/Living with Partner	852(57.18)	648(58.17)	204(54.25)	
Widowed/Divorced/Separated	553(37.11)	404(36.27)	149(39.63)	
Never married	85(5.71)	62(5.56)	23(6.12)	0.415
BMI (kg/m^2^)	30.65[27.70, 34.20]	30.60[27.60, 34.10]	30.80[28.10, 34.40]	0.232
24-hour total nutrient intake				
Total energy (kcal)	1675.00[1240.75, 2195.50]	1750.50 [1292.25, 2255.25]	1456.50[1088.5, 2007.0]	<0.001
Protein (g)	64.16 [46.50, 88.37]	66.66[48.85, 89.75]	58.67[42.10, 82.95]	<0.001
Carbohydrate (g)	200.69[148.80, 271.49]	204.80[153.79, 272.96]	186.19[133.62, 261.97]	<0.001
Dietary fiber (g)	14.30[9.60, 20.90]	14.80[10.00, 21.30]	12.80[8.30, 19.00]	<0.001
Total fat (g)	62.19[42.00, 90.87]	67.00[44.11, 93.93]	52.41[35.63, 77.96]	<0.001
Alcohol (g)	0.00[0.00, 0.00]	0.00[0.00, 0.00]	0.00[0.00, 0.00]	0.001
Laboratory tests				
RBC folate (nmol/L)	1260.00[908.25, 1727.50]	1290.00(927.25, 1730.00]	1195.00[877.25, 1720.00]	0.030
glycosylated hemoglobin (%)	5.80[5.50, 6.40]	5.80[5.50, 6.20]	6.00[5.60, 6.70]	<0.001
Albumin (g/L)	42.00[40.00, 44.00]	42.00[40.00, 44.00]	41.00[39.75, 43.00]	0.001
Serum creatinine (mmol/L)	80.44[68.07, 99.01]	79.56[67.18, 96.36]	83.98[69.62, 110.50]	<0.001
Hypertension	998(66.98)	736(66.07)	262(0.67)	0.197
Diabetes (%)	412(27.65)	269(24.15)	143(38.03)	<0.001
Asthma	220(14.77)	165(14.81)	55(14.63)	0.930
Arthritis	811(54.43)	581(52.15)	230(61.17)	0.002
Gout	132(8.86)	88(7.90)	44(11.70)	0.024
Angina	86(5.77)	57(5.12)	29(7.71)	0.061
Stroke	90(6.04)	52(4.67)	38(10.11)	<0.001
Liver disease	87(5.84)	58(5.21)	29(7.71)	0.073
Depression	400(26.85)	252(22.62)	148(39.36)	<0.001
Physical activity (%)				
Vigorous work activity	161(10.81)	141(12.66)	20(5.31)	<0.001
Moderate work activity	439(29.46)	363(32.59)	76(20.21)	<0.001
Vigorous recreational activity	113(7.58)	102(9.16)	11(2.93)	<0.001
Moderate recreational activity	558(37.45)	460 (41.29)	98(26.06)	<0.001
Sedentary(min)	360.00[240.00, 480.00]	360.00[240.00, 480.00]	360.00[240.00, 480.00]	0.270
Sleep time(h)	7.00[6.00, 8.00]	7.00[6.00, 8.00]	7.00[6.00, 8.00]	0.775
Smoking (%)	747(50.13)	554(49.73)	193(51.33)	0.591

### 3.2 Results of Lasso regression and logistic regression analysis

**Figure 1:**
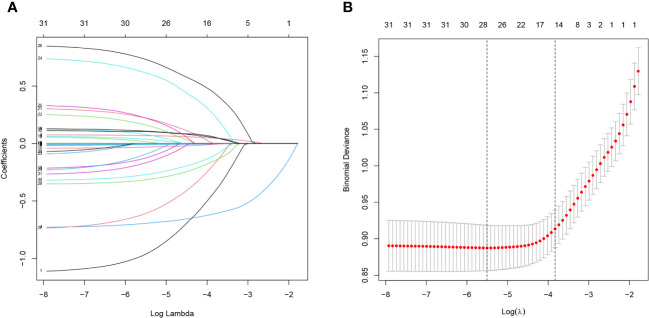
Lasso regression analysis of risk factors for cognitive dysfunction in abdominal obesity. **(A)** coefficient profile was created using the logarithmic (lambda) sequence, and the optimal lambda yielded non-zero coefficients. **(B)** Tenfold cross-validation was used to identify the ideal parameter (lambda) in the LASSO model utilizing minimal requirements. Plotting was done with the partial likelihood deviation (binomial deviation) curve in relation to log (lambda). A virtual vertical line at the optimal value was drawn using one SE of minimum criterion (the 1-SE criterion).

Lasso regression analysis was performed to identify optimal predictors (**Figure 1**). These predictors were then used in multivariate logistic regression analysis, which revealed that age (*p* < 0.001), sex (*p* < 0.001), education level (*p* < 0.001), 24-hour total fat intake (*p* < 0.001), RBC folate (*p* = 0.007), depression (*p* < 0.001), and moderate work activity (*p* = 0.008) were related with cognitive dysfunction in individuals with abdominal obesity with statistical significance (**Table 2**).

**Table 2:** Multivariate Logistic regression analysis of the risk of cognitive dysfunction in people with abdominal obesity.

Variable	Multivariate analysis	95%CI	P-value
	Odds ratio		
Age	1.07	1.04-1.10	<0.001
Sex			
Male	Reference		
Female	0.36	0.23-0.55	<0.001
Education			
Less than 9th grade	Reference		
9-11th grade	0.54	0.28-1.05	0.070
High school graduate	0.24	0.13-0.45	<0.001
Some college graduate	0.10	0.05-0.20	<0.001
College graduate or above	0.08	0.04-0.18	<0.001
Total fat	0.99	0.98-0.99	<0.001
RBC folate	1.00	0.99-1.00	0.007
Depression			
Yes	2.34	1.56-3.53	<0.001
No	Reference		
Moderate work activity			
Yes	0.56	0.36-0.86	0.009
No	Reference		

### 3.3 Development of nomogram

**Figure 2:**
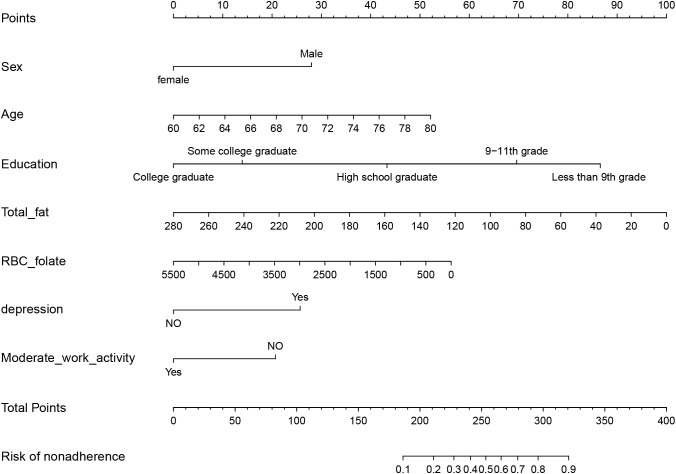
Nomogram prediction model for cognitive dysfunction in abdominal obesity.

According to the findings of Lasso and logistic regression analyses, a prediction model consisting of seven predictors (age, sex, education, total fat intake, RBC folate, depression, and moderate work activity) was constructed. This model was represented as a nomogram (**Figure 2**). According to different variables, a vertical line is drawn at the top of the nomogram to obtain the corresponding score. The scores of each variable are added together to obtain the total score, and the corresponding total risk score is obtained at the bottom of the nomogram. For example, if a 72-year-old male graduated from high school at a young age with a total fat intake of 80g in 24 hours, a red blood cell folate concentration of 1500 nmol/L, no history of depression, and no moderate physical activity, the total score is 237.5 points (28 points for gender, 31 points for age, 44 points for high school graduation, 72 points for total fat intake in 24 hours, 42.5 points for red blood cell folate concentration, 0 point for history of depression, and 20 points for moderate physical activity). For the mentioned patient, the total score is 237.5, and the probability corresponding to this total score on the nomogram will be between 0.3 and 0.4. The probability of cognitive dysfunction in this elderly person is estimated to be between 30% and 40%.

### 3.4 Assessment of predictive nomogram

**Figure 3:**
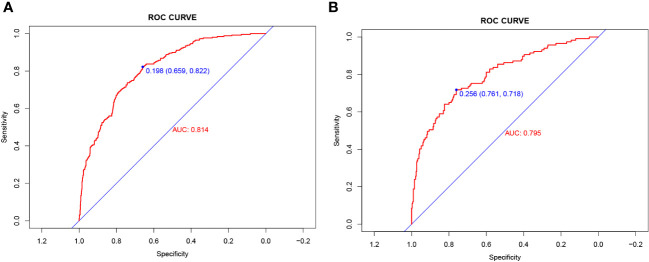
**(A)** Nomogram ROC curves generated from the training dataset. **(B)** Nomogram ROC curves generated using the validation dataset.

**Figure 4:**
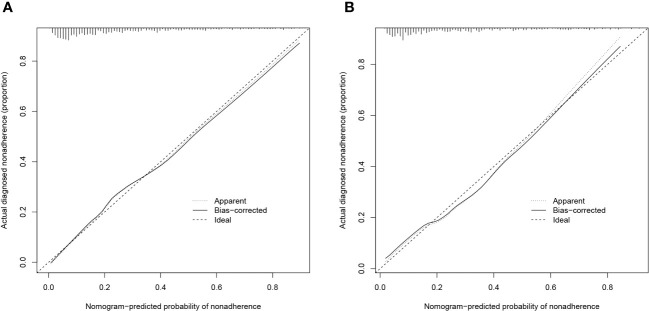
**(A)** Calibration plot for the training dataset. **(B)** Calibration plot for the validation dataset.

**Figure 5:**
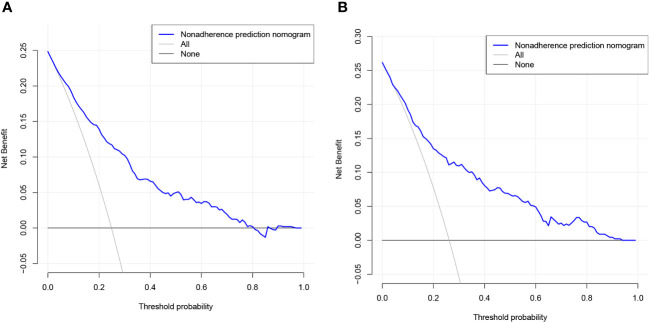
**(A)** DCA curves for the training dataset. **(B)** DCA curves for the validation dataset.

The validity and discriminative ability of the nomogram were evaluated using various metrics. The C-index for the training set was 0.814 (95% CI: 0.875-0.842), indicating good discriminability. The C-index for the validation set was 0.805 (95% CI: 0.758-0.851), further confirming the model’s discriminative power. The sensitivity, specificity, and AUC of the training group were 0.814, 0.659, and 0.822, respectively. For the validation group, the AUC was 0.795, specificity was 0.761, and sensitivity was 0.718 (**Figure 3**). These results demonstrated the strong discriminative and predictive capabilities of the nomogram. The calibration curves for both sets were nearly straight lines with a slope of 1, indicating good agreement between predicted probabilities and actual outcomes (**Figure 4**). DCA was applied to assess the clinical validity of the model (**Figure 5**). The DCA showed that the prediction model provided net benefits for both the training and validation sets, indicating superior net benefits and prediction accuracy of the nomogram model.

## 4 Discussion

Over the past few years, there has been a consistent rise in the prevalence of obesity (21), highlighting a concerning trend in public health. This alarming rise in obesity rates has captured the attention of individuals, healthcare professionals, and governments alike. The prevalence and mortality of dementia are also increasing due to aging and population growth (22). One study, which followed up individuals for 42 years, found that obesity was associated with dementia later in life (23). Other studies have investigated the relationship between pre-dementia BMI and subsequent dementia development, suggesting it may be a predictor (24). Obesity can impair cognitive functions such as memory, speech learning, and executive ability (25). Some studies have shown that not only the volume but also the location of fat deposition is associated with disease, particularly the deposition of visceral fat in the abdomen, which plays a significant role independent of total fat (26). A Mendelian randomized study involving nearly 10,000 Asian individuals found that visceral fat and BMI may contribute to cognitive decline. Each 0.27 kg increase in abdominal fat was equivalent to 0.7 years of cognitive decline (27). Another cohort study, which included 9,189 participants and excluded those with clinically diagnosed cardiovascular disease while correcting for confounding factors such as educational attainment, arrived at a similar conclusion. It was found that for every 9.2% increase in body fat percentage or 36 milliliters of visceral fat, there was an accelerated aging of 1 year in terms of corresponding cognitive functions (28). Therefore, it is crucial to understand the risk predictors of cognitive dysfunction in abdominal obesity and take steps to reduce its incidence.

The results of this study revealed that age, sex, education, total fat intake, RBC folate, depression, and moderate work activity were important predictors of cognitive dysfunction in individuals with abdominal obesity. Age is a significant factor affecting cognition (29), which gradually declines with increasing age. Dario Bachmann et al. (30) showed that age is a major factor in cognitive variability, significantly correlating with white matter hyperintensities (WMH) and lateral ventricular volumes. Age also showed a strong correlation with cortical thickness in a large neocortical ROI (NEOcomp ROI) and hippocampal volume, leading to cognitive decline.

Our study found that males were more likely to have cognitive dysfunction. Research shows that men are more prone to developing mild cognitive impairment (31) compared to women. In individuals with the apolipoprotein E genotype ϵ2/ϵ3, males have a higher risk of developing Alzheimer’s disease than females (32). Among older adult males, cognitive dysfunction is more likely to occur in those who are older, have higher education levels, are nulliparous, have depressive symptoms, and are socially inactive (33). Therefore, interventions should be tailored to different populations.

We also found that as education levels increased, the odds of cognitive dysfunction decreased in individuals with abdominal obesity. The mechanisms by which education improves cognitive abilities have not been fully understood yet. It may be related to the quality of education, occupational complexity, and participation in cognitive intellectual activities. Additionally, individuals with lower education levels may have less knowledge about diseases and receive less health education. Higher education and early access to education may, therefore, be beneficial in delaying cognitive decline in later life (34).

Dietary nutrient intake also has an impact on cognitive function (35). However, this study found that 24-hour total fat consumption was a risk indicator for cognitive dysfunction in individuals with abdominal obesity. These findings may be associated with the type of fat consumed; for example, consuming a high amount of polyunsaturated fatty acids may reduce the risk of cognitive dysfunction (36). Monounsaturated and polyunsaturated fatty acids can help lower LDL cholesterol levels and increase HDL cholesterol level (37). A prospective observational study by Gustafson et al. (38) also found that individuals who consumed higher amounts of total polyunsaturated fat had a lower risk of Alzheimer’s disease and dementia. Therefore, further investigation is needed to understand the relationship between different types of dietary fat and cognitive function in obese individuals with abdominal obesity.

Furthermore, there is a significant link between folic acid intake and cognitive performance, with research showing that higher folic acid intake is associated with improved cognitive function when vitamin B12 intake is normal (39), which was consistent with our findings. Folate deficiency can lead to elevated homocysteine levels and affect cognitive function (40). In a controlled study, participants who received 800ug of oral folic acid daily experienced a 53.9% increase in serum folate concentration after three years, and improvements in cognitive abilities such as memory, information processing speed, as well as sensory motor speed were much better compared to the placebo group (41). Therefore, folic acid supplementation may help delay cognitive deterioration.

Depression is common in individuals with cognitive dysfunction. A 30-year study found that individuals with mental disorders in adolescence were more likely to develop dementia later in life compared to those without mental disorders (42). Additionally, Holly Elser et al. found that patients with depression had an increased cumulative risk of dementia compared to controls, with an overall hazard ratio of 2.41. A diagnosis of depression at an early, middle, or late age was linked to a higher dementia risk (43). Thus, paying attention to mental health at an early age may reduce or delay the burden of dementia later in life.

Moderate work activity, defined as work involving moderate physical activity like brisk walking or lifting light objects for 10 minutes continuously, resulting in a mild increase in respiration or heart rate, is consistent with most studies showing that moderate physical activity reduces the risk of dementia (44). Encouraging individuals with abdominal obesity to engage in moderate physical activity at work is therefore important.

The nomogram has been extensively used in various clinical trials and is a commonly employed predictive model in clinical practice (45, 46). However, no nomogram has been reported to predict the development of cognitive dysfunction based on data from individuals with abdominal obesity. This study used nine predictors preselected by LASSO regression analysis to build a nomogram for predicting the probability of cognitive dysfunction in individuals with abdominal obesity. The nomogram models were well calibrated and clinically relevant, enabling effective identification of cognitive dysfunction in individuals with abdominal obesity.

Although the C-index, ROC curves, calibration curves, and clinical utility of the nomogram have been well validated, the study does have a few limitations. Firstly, it is a cross-sectional study with potential selection bias, thus requiring validation through prospective and multi-center studies. Secondly, the diagnosis of cognitive impairment was assessed using various scales, which may introduce bias.

## 5 Conclusion

This study employs Lasso regression and multivariable logistic regression to select potential predictive factors. Ultimately, seven predictive variables, including age, sex, education, total fat intake, RBC folate, depression, and moderate work activity, are integrated into the nomogram. The data for these variables are readily accessible, and the predictive model is easy for clinical use. It is clinically significant for primary healthcare workers or primary care physicians to rapidly assess the risk of cognitive impairment in individuals with abdominal obesity. This helps control the development of cognitive impairments, thus reducing the socioeconomic burden and caregiving pressure.

## Data availability statement

The datasets presented in this study can be found in online repositories. The names of the repository/repositories and accession number(s) can be found in the article/supplementary material.

## Ethics statement

The studies involving humans were approved by the National Center for Health Statistics Ethics Review Board. The studies were conducted in accordance with the local legislation and institutional requirements. Written informed consent for participation was not required from the participants or the participants’ legal guardians/next of kin in accordance with the national legislation and institutional requirements.

## Author contributions

CL: Data curation, Formal analysis, Investigation, Project administration, Writing – original draft, Writing – review & editing. GW: Data curation, Formal analysis, Investigation, Project administration, Writing – original draft, Writing – review & editing. YC: Methodology, Resources, Visualization, Writing – review & editing. HX: Methodology, Resources, Visualization, Writing – review & editing. JC: Software, Writing – review & editing. XYZ: Conceptualization, Investigation, Project administration, Supervision, Writing – review & editing. YL: Validated, Writing – review & editing. ML: Validated, Writing – review & editing. RZ: Conceptualization, Investigation, Project administration, Supervision, Writing – review & editing. XFZ: Software, Writing – review & editing.
